# Vivisection in the District of Columbia

**Published:** 1897-07

**Authors:** 


### VIVISECTION IN THE DISTRICT OF COLUMBIA.

At the late Jubilee Meeting of the American Medical Association Dr. H. C. Wood, of Philadelphia, made a vigorous speech protesting against the passage of the bill now pending before the United States Senate to "regulate the practice of vivisection in the District of Columbia." Dr. W. W. Keen, of Philadelphia, did likewise, and in his eloquent address on Surgery said: "Bacteriology would not now exist as a science, nor would accurate modern surgery and a large part of modern medicine be possible, had experiments upon animals been prohibited, as some zoöphilous men and women who love dogs better than men and women, and even little children, desire." The following resolution was adopted by the Association with great enthusiasm:

*Whereas*, We believe the passage of Senate Bill 1063 would seriously interfere with the progress of practical medicine, and therefore be a public calamity; therefore be it

*Resolved*, That the American Medical Association, with full knowledge of the contents of said bill, most earnestly protests against its enactment.

Dr. Wood also stated that if the physicians of the United States would make this a personal matter and present their individual protests to their respective congressmen, their "influence in crushing the measure would be irresistible."

It is to be hoped that this will be speedily done.

---

The proposed consolidation of Bellevue Hospital Medical College with the University Medical College has been declared off, owing to difficulties in arranging the details of amalgamation. The selection of a faculty for the future school is said to have been the principal obstacle to the union. It is very much to be regretted that the union could not be effected on some satisfactory basis. So the two schools will continue to do business, as heretofore, at the old stands.

